# Sensorimotor Stabilization Exercises With and Without Behavioral Treatment in Low Back Pain: Feasibility and Effects of a Multicenter Randomized Controlled Trial

**DOI:** 10.1016/j.arrct.2025.100430

**Published:** 2025-01-27

**Authors:** Tilman Engel, Daniel Niederer, Adamantios Arampatzis, Winfried Banzer, Heidrun Beck, Philipp Floessel, Thore Haag, Steffen Mueller, Marcus Schiltenwolf, Hendrik Schmidt, Christian Schneider, Dirk Stengel, Josefine Stoll, Pia-Maria Wippert, Frank Mayer

**Affiliations:** a University Outpatient Clinic, Sports Medicine & Sports Orthopedics, University of Potsdam, Potsdam, Germany; b Division of Preventive and Sports Medicine, Institute for Occupational Medicine, Social Medicine and Environmental Medicine, Goethe-University Frankfurt, Frankfurt am Main, Germany; c Department of Training and Movement Sciences, Humboldt-Universität zu Berlin, Berlin, Germany; d University Hospital Carl Gustav Carus at Technical University Dresden, Dresden, Germany; e Orthopädiezentrum Theresie, München, Germany; f Department of Computer Science – Therapeutic Sciences, Physiotherapy, Exercise Science and Applied Biomechanics, Trier University of Applied Sciences, Trier, Germany; g Pain Management, Center of Orthopedics and Trauma Surgery, Conservative Orthopedics and Pain Management, Heidelberg University Hospital, Heidelberg, Germany; h Julius Wolff Institute, Berlin Institute of Health @ Charité-Universitätsmedizin Berlin, Berlin, Germany; i BG Kliniken - Klinikverbund Der Gesetzlichen Unfallversicherung gGmbH, Berlin, Germany; j Medical Sociology and Psychobiology, University of Potsdam, Potsdam, Germany; k Faculty of Health Sciences Brandenburg (joint Faculty of the University of Potsdam, the Brandenburg Medical School Theodor Fontane and the Brandenburg University of Technology Cottbus – Senftenberg), Cottbus, Germany

**Keywords:** Adherence, Back pain, Behavioral, Behavioral intervention, Disability, Exercise, Exercise intervention, Exercise therapy, Feasibility, Functional assessment, Pain intensity, Peak force, Rehabilitation, Sensorimotor, Stabilization

## Abstract

**Objectives:**

To investigate the feasibility and effects of a sensorimotor stabilization exercise intervention with and without behavioral treatment in nonspecific low back pain.

**Design:**

A three-armed multicenter randomized controlled trial.

**Setting:**

Five study sites across Germany (3 orthopedic university outpatient clinics, 1 university sports medicine department, and 1 clinical institution).

**Participants:**

Six hundred and sixty-two volunteers (N=662) (59% females, age 39±13y) with low back pain.

**Interventions:**

Sensorimotor training (SMT), sensorimotor training with behavioral therapy (SMT+BT), and usual care group (UCG; continuation of the already ongoing individual treatment regime). Intervention groups performed a 12-week (3wk center-based, 9wk home-based) program.

**Main Outcome Measures:**

Adherence, dropout rates, adverse events, and intervention effects on pain intensity, disability, and trunk torque (gain scores, repeated measures analysis of variance, α-level<0.05).

**Results:**

In total, 220 participants received SMT, 222 received SMT+BT, and 170 were analyzed as UCG. Dropout rates were 10% for SMT and SMT+BT at week 3, 31% and 30% at week 4, and 49% and 50% at week 12. Adherence rates above 80% were reached in both interventions; 134 adverse events occurred. Intervention effects compared to UCG were found for pain intensity (SMT, *P*=.011, effect size d=0.41), disability (SMT+BT, *P*=.020, d=0.41), and peak torque (SMT, *P*=.045, d=0.38; SMT+BT, *P*=.019, d=0.44), with overall small effect sizes.

**Conclusions:**

Participants were highly adherent to the sensorimotor exercise, but showed increased dropout rates, particularly during home-based training. Both interventions proved to be feasible, and although only SMT showed an increased effect on pain intensity compared to UCG, the SMT+BT showed positive effects on disability. Both interventions led to increases in strength, indicative of a neuromuscular adaptation.

Structured exercise interventions are the key component of current first-line treatments of nonspecific low back pain.^1^ The most effective exercise types are likely those that target musculoskeletal control by affecting afferent sensory input, central nervous system integration, and motor control to ensure functional dynamic joint stability during perturbative situations addressing motor function,^2^ such as stabilization, motor control, and sensorimotor exercises.^3^^,^^4^ Despite this likely superior effectiveness to other exercises, interventional effects of stabilization exercises are often only low to moderate and heterogeneous.^5^^,^^6^ The effects, and thus also the likely superiority to other treatments, are mostly evidenced by key outcomes such as pain, physical function and disability, and quality of life solely.^7^ Effects on other objective functional outcomes^8^ remain largely unknown.

In addition, numerous contributors are suggested to affect the effects reachable by exercise in nonspecific low back pain. Psychological interventions such as (cognitive) behavioral therapy are most effective when delivered in conjunction with structured exercises.^9^ Furthermore, a key component of exercise effects in low back pain is treatment feasibility in the sense of low dropout rates as well as adherence to the scheduled exercises.^3^^,^^4^^,^^10^ Exercise adherence is the major link between intervention and training success.^11^, ^12^, ^13^, ^14^

In the population of people with less severe low back pain, treatment effects of sensorimotor/stabilization exercises with and without behavioral treatments are scarcely investigated. This leads to a research deficit in robust evidence on the effect and feasibility of a large population of people affected by nonspecific low back pain. Therefore, the aim of this study was to analyze whether sensorimotor exercises with and without behavioral treatment are feasible in terms of adherence to a scheduled therapy regimen and whether both interventions are more effective than usual care. It is hypothesized that the sensorimotor treatment and the combined sensorimotor and behavioral treatment are feasible in terms of adherence to a scheduled therapy regimen, and that both interventions are more effective than a usual care comparator.

## Methods

### Study design and ethics

This single-blinded, 3-armed multicenter randomized controlled trial (German Clinical Trial Register: DRKS00004977) was conducted at 5 study sites (3 orthopedic university outpatient clinics, 1 university sports medicine department, and 1 clinical institution) of the research network “Medicine in Exercise Science (MiSpEx).” Ethical approval was provided by the local institutional ethics review board of the corresponding study center (number 36/2011), as well as by secondary ethical approval at all sites.

### Participants

Participants were recruited during consulting hours at orthopedic outpatient clinics, as well as through advertising (university sports, physiotherapy facilities, print media). Inclusion criteria were as follows: age between 18 and 65 years, non-acute (low) back pain (>20 on a 100mm Visual Analog Pain Scale), and German language skills. Exclusion criteria were as follows: acute infections, pregnancy, inability of upright standing/getting up from a lying position, inability to fill out questionnaires, illnesses/conditions contraindicating physical activity, and acute back pain that started up to 7 days before study inclusion. All eligible participants subscribed to informed consent before their enrolment in the study. All volunteers underwent a clinical examination. The sample size calculation (α≤0.05; 1−β=0.95, dropout 30%, G*Power^a^) was based on a pilot investigation (unpublished material) for the outcome pain, yielding a conservative effect size of f=0.25.

### Randomization

Participants were randomly allocated to the 3 study groups (with block sizes of 9 participants) by the study coordinator, subsequent to all assessments of day 1. Randomization lists were provided by the central study organization and concealed by each center's study coordinator. Clinical personnel and investigators were blinded to participants’ group allocation for the whole study duration. Study volunteers and therapists were unblinded.

### Interventions

**Fig 1:**
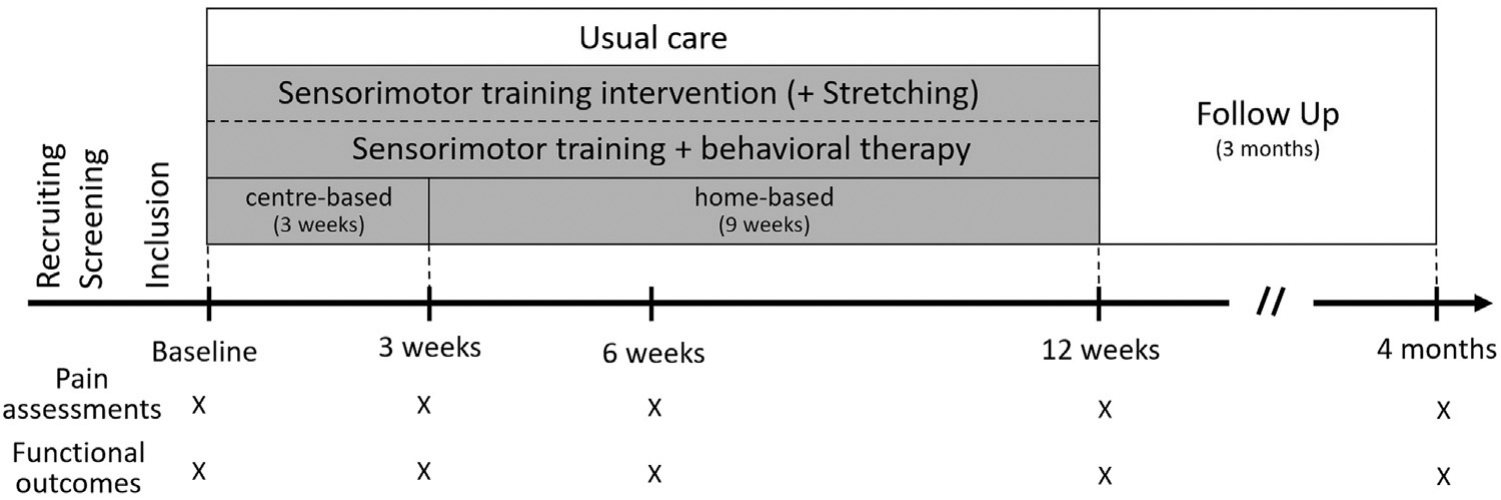
Study time bar.

Two different 12-week intervention programs (sensorimotor training(SMT) and sensorimotor training with behavioral therapy modules [SMT+BT]) were compared to a usual care group (UCG; continuation of the already ongoing individual treatment regime). A structured and supervised 3-week center-based training was followed by a 9-week home-based training (fig 1). The center-based training was conducted in groups of 2-5 participants under the supervision of an experienced sports therapist at the corresponding clinical training centers. During home-based training, participants were advised to continue their training program based on a standardized training prescription.

A training frequency of 3 sessions per week was scheduled, both during the center-based and home-based training phase. Each training session lasted 30-40 minutes. Training sessions with corresponding levels of difficulty were documented over the entire intervention period in a training log.

**Fig 2:**
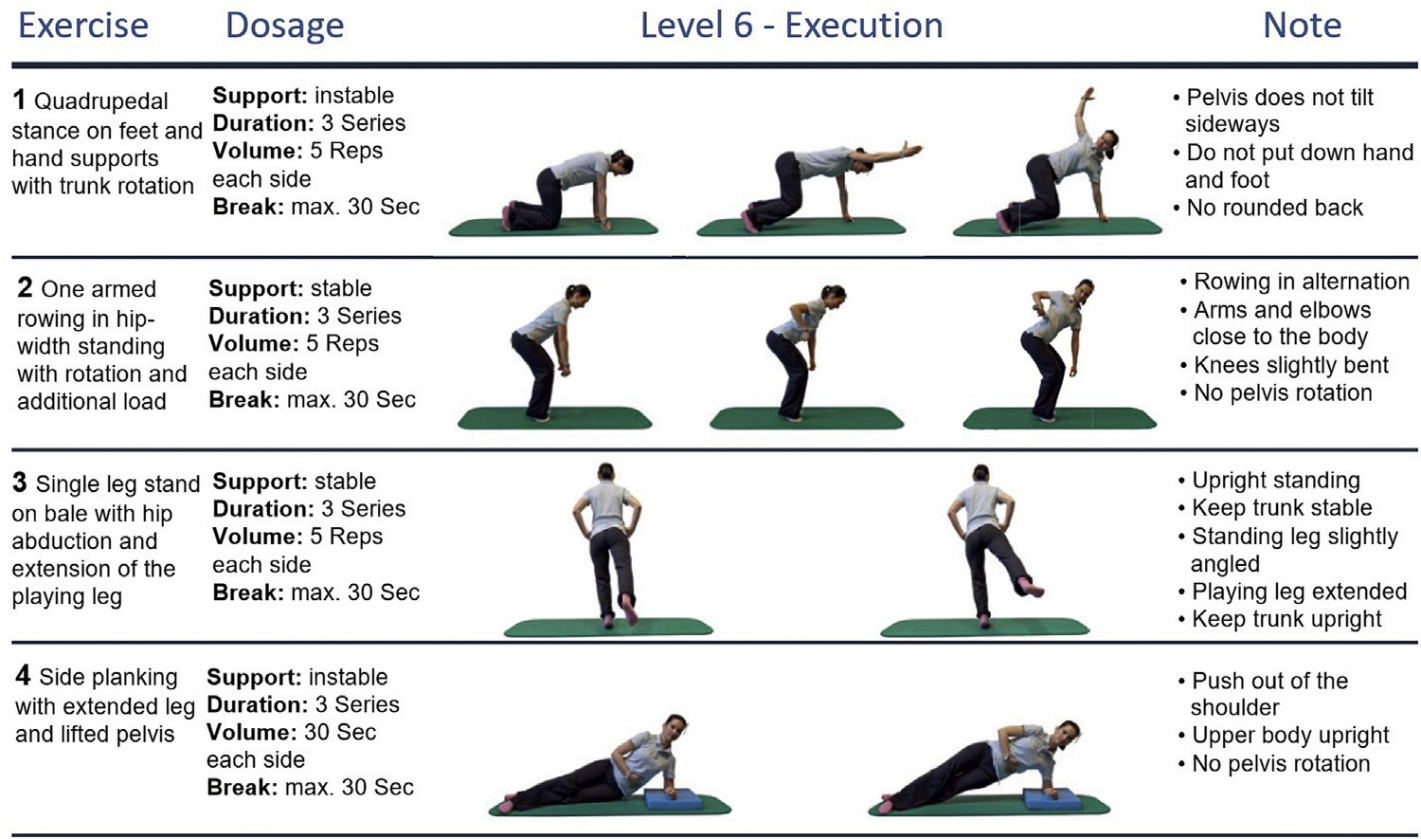
Exemplary exercises of the sensorimotor training program at difficulty level 6 (of maximum 12 level) with 2 exercises performed in standing position and 2 in quadrupedal/side planking position.

Both groups’ exercise programs consisted of 4 different core-specific stabilization exercises. Two of the exercises were performed in standing position (1-leg-stance and front rowing position), and another 2 in quadrupedal or side planking position. Each of the exercises consisted of 12 difficulty levels, gradually increasing the sensorimotor demand by altered support surfaces (varying foam pads), foot positions (eg, standing on the rear or forefoot), or additional loads and limb movements. Examples of exercises in the program are shown in figure 2. Individualized entry training levels were determined by the sports therapists and adapted over the center-based period (by increasing/decreasing the level). After the supervised training phase, starting levels and planned increments for the home-based phase were established in a shared process by the sports therapist and participant.

After the exercise program, the SMT group performed standardized stretching exercises (exclusively of the lower and upper extremities; to match the overall training session duration), whereas the SMT+BT group received 3 additional behavioral modules. The latter consisted of a cognitive distraction task during exercises, a body scan unit directly after the exercises, and psychoeducation.^15^^,^^16^ Participants of the UCG did not receive any scheduled exercise intervention within the study but were instructed to maintain their regular exercise levels as well as their clinical back pain treatment strategies without further standardized advice from the study personnel. Participants of the intervention groups were also free to continue with their regular clinical back pain treatment strategies. All participants of the intervention groups received printed information about the exercises and a training log to document their performed exercises (during center-based and home-based training).

### Testing setup and outcomes

Each participant was scheduled for 5 measurement visits (baseline, 3, 6, 12, and 24wk [fig 1]). During each visit, pain intensity and pain-related disability were reported by the “Chronic Pain Grade questionnaire”^17^ using the subscales “characteristic pain intensity” (CPI: 0 = “no pain” to 100 = “the worst pain imaginable”), and “subjective disability score” (DS: 0 = “no disability” to 100 = “I was incapable of doing anything”) on average over the preceding 3 months. For CPI, an average score of “pain right now,” “worst pain in the past 3 months,” and “average pain in the last 3 months” is calculated. For DS, an average score of pain-related disability during “daily activities,” “social activities,” and “work activities” is used.^17^

Subsequently, motor function was assessed based on a previously established set of functional outcome measures for low back pain.^8^ The measurements were conducted in standardized order: (1) postural control; (2) jumping assessments (prefatigue task); (3) strength testing; and (4) repeated jumping assessment (postfatigue task).

Postural control was measured in an upright unipedal stance (no shoes, hands at hips, eyes open) in 2 trials for each leg (randomized order) using identical mobile balance boards across all centers (Wii Balance Board^b^, Humac Balance^c^). The outcome measure was the trace length (mm) of the excursion of the center of pressure (CoP) for 10 seconds.

Jumping performance was measured by bipedal countermovement jumps (CMJ; upright standing; hands at hips; 90° knee flexion) in 3 trials using force plates (1000Hz; AMTI Force plate^d^, Kistler force plate^e^). The outcomes were flight time (CMJ flight time; [ms]) and peak force during liftoff (CMJ peak force, [N]).

Maximum trunk force was measured either in isokinetic (3 centers) or isometric (2 centers) conditions by dynamometry (CON-TREX MJ/TP 1000^f^; Isomed 2000^g^; Tergumed Plus Flexion^h^; Schnell m3 diagnos+^i^). Isokinetic trunk force (60°/s; 55° range of motion) was assessed during 5 maximal effort repetitions (after 30 submaximal repetitions for warm-up). Isometric trunk force (3s; flexion/extension) was measured during 3 maximal effort repetitions (after 3 submaximal repetitions for warm-up). Participants were verbally encouraged to elicit maximum effort, in a standardized manner. The outcome measure was maximum torque (Nm) during trunk extension/ flexion tasks.

Jumping tasks in a fatigued state (assessed only by study centers with isokinetic testing) were performed immediately after an additional set of 26 repetitions (approximately 1min) of trunk flexion/extension under identical conditions as before.

Data of the CoP trace length (MATLAB-R2019b^j^) were averaged across all 4 unipedal stances. Flight time and peak force (MATLAB-R2019b^j^; Excel-2016^k^) were averaged across all 3 CMJ tasks. Maximum torque outcome was based on the average values of the 3 highest peak torques, both for isokinetic and isometric testing (Excel-2016^k^).

### Statistical analysis

All data were documented in a case report form unless captured digitally by measurement devices. Data were manually transferred into a database for further statistical analysis, with an initial plausibility check (range check, back-tracking a randomly drawn subsample to raw data, recalculation, and, where required, deletion of implausible values and outliers).

The baseline characteristics of participants (anthropometrics, pain intensity/disability, functional outcomes) were compared by 1-way analysis of variance (ANOVA, α-level cutoff <0.05, post hoc testing via *t* tests for independent samples; SPSS Statistics Version 28^l^).

The feasibility of exercise interventions and testing setup was assessed by intervention adherence (sessions per week and percentage of scheduled sessions of active participants) via training logs, dropout rates related to the intervention (termination of active training participation for center-based, home-based, and whole intervention phase), dropout rates related to the measurement visits (serious), adverse events, and training level progression (increase in difficulty level over time). A survival analysis with a corresponding plot was performed to assess training dropout rates over the intervention periods. Further, the numbers of adverse events were compared between the study groups by chi-square analysis.

Intervention effects were estimated by pain-related (pain intensity/disability) and functional outcomes (trunk force, CoP, CMJpre/post) using descriptive statistics (mean ± SD, ranges, 95% confidence interval) based on the full dataset (containing all available data for each measurement visit) . After checking the underlying data for assumptions to perform the corresponding test (normality of the variances, variance homogeneity), gain score analysis was performed for baseline to postintervention (12wk) by one-way (ANOVA, α-level cutoff <0.05, post hoc testing via *t* tests for independent samples; SPSS Statistics Version 28^l^) based on a complete case dataset; corresponding data were presented as violin plots. The associated effect sizes (Cohen's d standardized mean differences) were calculated for intervention groups in comparison to UCG. To account for differences in isokinetic force assessments between centers (isometric/isokinetic; different manufacturers), torque data were analyzed as percentage change normalized to baseline.

## Results

### Participant flow

**Fig 3:**
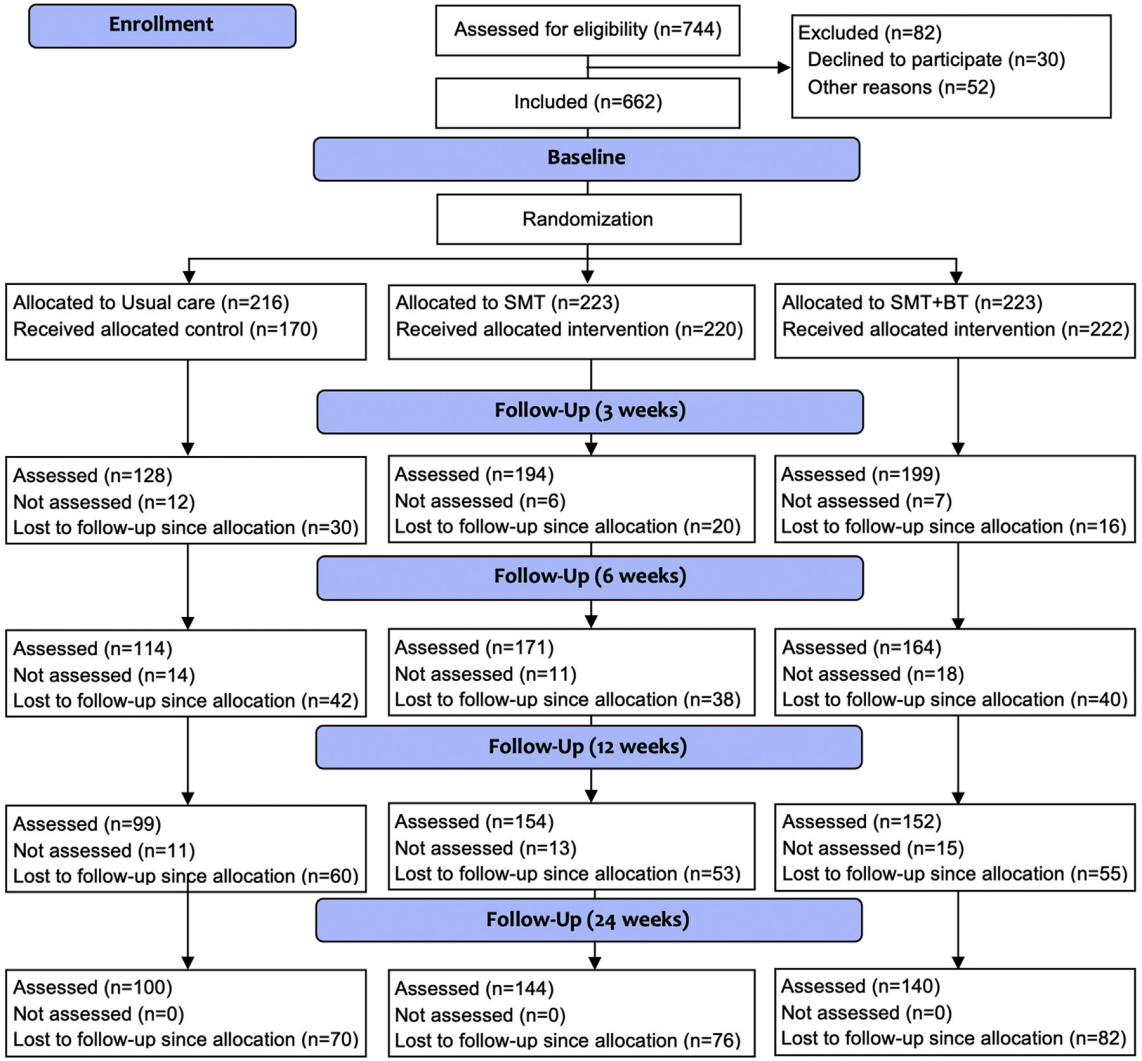
Participant's flow chart (UCG: usual care, SMT: sensorimotor training, SMT+BT: sensorimotor training + behavioral therapy).

The study and participant flow are displayed in the CONSORT chart in figure 3. Because of a failure in the randomization process in one of the study centers, 46 participants were allocated to the UCG, instead of being randomized to either the usual care or one of the intervention groups. To avoid potential bias arising from this failure, all affected participants were excluded before the analysis. During the post-visits, the dropout rate for the SMT group was 9% after 3 weeks, 17% after 6 weeks, 24% after 12 weeks (end of intervention), and 35% after 24 weeks (follow-up); in the SMT+BT group, the corresponding values were 7%, 18%, 25%, and 37%, and in the UCG 18%, 25%, 35%, and 41% (fig 3). Most of the participants withdrew their consent without providing a reason. For those where specific information is available, 70% dropped out because of “lack of time,” 10% because of a “medical condition,” and 20% because of other various reasons (eg, pregnancy, change of residence, family-related issues, lack of interest). There were no serious adverse events, but 134 adverse events by 120 participants (increase in pain, to the extent that individuals were not able to participate in functional assessments) were reported. No differences were found for frequencies of adverse events between study groups (x^2^=0.449, *P*=.799). A comparison of people who completed the intervention to those who dropped out revealed no statistically significant difference in the corresponding baseline values of all outcomes (*t* tests for independent samples, α-level cutoff <0.05).

### Baseline characteristics

Baseline characteristics for the whole sample and separated by group allocation are provided in table 1, including the results of statistical testing for differences between groups.

**Table 1:** Participant's anthropometrics, pain characteristics, and functional outcomes at baseline

Domains and Outcomes		Total Sample		Usual Care		SMT		SMT+BT	
		N	Mean ± SD	N	Mean ± SD	N	Mean ± SD	N	Mean ± SD
Anthropometrics	Sex/sex (% females)	612	59	170	58	220	57	222	60
	Age* (y)	612	40±13	170	37±12	220	41‡±12	222	41‡±13
	Height* (cm)	612	173±9	170	174±9	220	173±9	222	173±9
	Weight* (kg)	612	73±14	170	72±14	220	72±14	222	73±14
Pain	Characteristic pain intensity (Score 0-100)	592	32±20	162	27±20	212	35‡±20	218	33‡±20
	Pain-related disability (Score 0-100)	586	19±22	163	14±19	210	20‡±21	213	22‡±23
Functional tests	Isokinetic trunk extension† (Nm)	355	202±87	81	199±77	133	196±84	141	209±96
	Isokinetic trunk flexion† (Nm)	358	128±50	84	136±55	133	125±49	140	127±49
	Isometric trunk extension† (N)	225	505±449	79	527±457	74	515±444	72	470±453
	Isometric trunk flexion† (N)	222	298±141	76	310±139	74	303±130	70	281±154
	CoP trace length (mm)	515	338±102	153	323±89	181	333±100	181	354±113
	CMJpre peak force (N)	447	1454±347	112	1461±325	160	1433±347	175	1471±363
	CMJpre flight time (ms)	566	420±74	157	430±72	200	422±72	209	410±76
	CMJpost peak force (N)	456	1518 ±356	121	1519±325	162	1521±366	173	1514±369
	CMJpost flight time (ms)	544	422±73	154	431±71	190	425±70	200	413±75

NOTE. Displayed are the means and standard deviations (mean ± SD).

Abbreviations: CMJpost, countermovement jump after fatigue task; CMJpre, countermovement jump before fatigue task; CoP, center of pressure; N, number of included participants; SMT, sensorimotor training (+stretching); SMT+BT, sensorimotor training+behavioral therapy.

⁎ For missing information at baseline measurement, data of measurement at end of the center-based training were taken.

† Trunk force during extension and flexion was assessed either isokinetically or isometrically, depending on the study center.

‡ Statistically significant differences compared to usual care; ANOVA post hoc testing (*P*<.05).

### Feasibility of the intervention

Full data training documentation was available in SMT for 88% and SMT+BT for 91% of participants. For the remaining participants, training data could not be analyzed because of the loss of the training records (by participants or training center).

**Fig 4:**
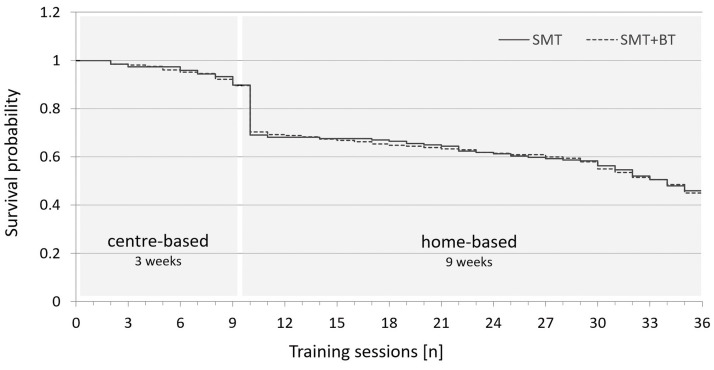
Survival plot of performed training sessions within the sensorimotor training (SMT) and the sensorimotor training with behavioral therapy (SMT+BT).

The dropout rate during center-based phase was 10%, both for SMT and SMT+BT. During the home-based phase, the dropout increased from 31% in SMT and 30% in SMT+BT (4wk) to 49% in SMT and 50% in SMT+BT (12wk). Training dropout by sessions assessed by survival analysis is displayed in figure 4.

Overall, during the center-based phase, participants performed in both SMT and SMT+BT groups in a mean of 2.6±0.6 sessions per week, and during the home-based phase, 2.5±0.7 in SMT and 2.5±0.8 in SMT+BT. Adherence rates for SMT resulted in 88% for the center-based phase and 84% for the home-based training phase, and for SMT+BT in 87% and 82% correspondingly.

Mean training level progression was 0.7 levels per week, leading to an average increase of 8.1 ± 3.0 levels at the end of the 12-week training intervention for both SMT and SMT+BT.

### Feasibility of the testing setup

In 81% of all cases, the scheduled functional testing battery was carried out completely. Reasons for limited test eligibility (determined by the physician before each visit) were clinical conditions, such as ankle sprains, knee, injuries, acute back pain, high blood pressure, or muscle soreness.

#### Intervention effects

**Fig 5:**
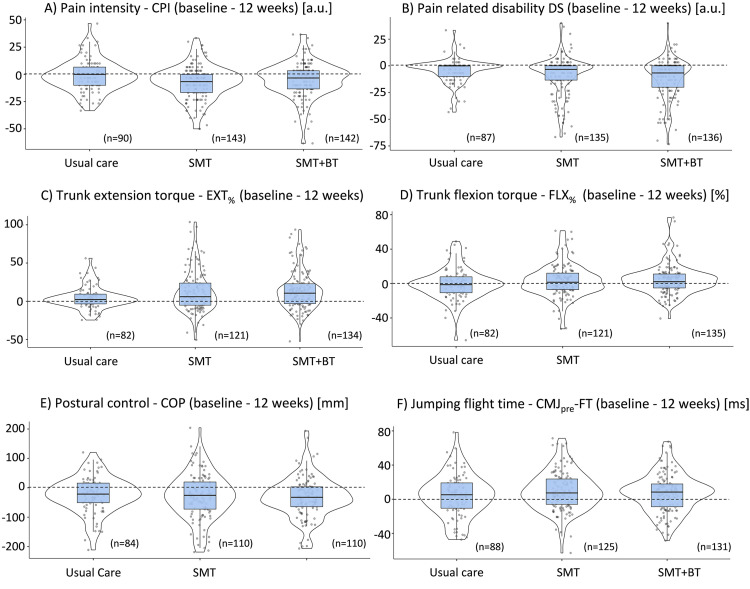
Violin plots of pain ratings and selected functional outcomes for gain scores (differences) from baseline to 12 weeks. Individual data (dots); kernel density estimator smoothed probability density of the data (curves); median (horizontal lines); interquartile ranges and whisker bars (vertical lines); 0 line (dotted horizontal line); CMJpre-FT, countermovement jump flight time before fatigue task; CoP, center of pressure trace; CPI, characteristic pain intensity; DS, disability score; EXT% and FLX%, combined isokinetic and isometric extensor and flexor peak torque changes in percentage; SMT, sensorimotor training; SMT+BT, sensorimotor training+behavioral therapy.

The gain score analysis (changes from baseline to postintervention at week 12) of the pain outcome measures revealed a statistically significant between-group difference (ANOVA: F(2, 372)=4.308, *P*=.014) for CPI between UCG and SMT (*P*=.011, effect size d=0.41), but not between UCG and SMT+BT (*P*=.258, d=0.23), and for pain-related disability (ANOVA: F(2, 355)=3.756, *P*=.024) between UCG and SMT+BT (*P*=.020, d=0.41), but not for UCG and SMT (*P*=.223, d=0.28) (fig 5).

**Fig 6:**
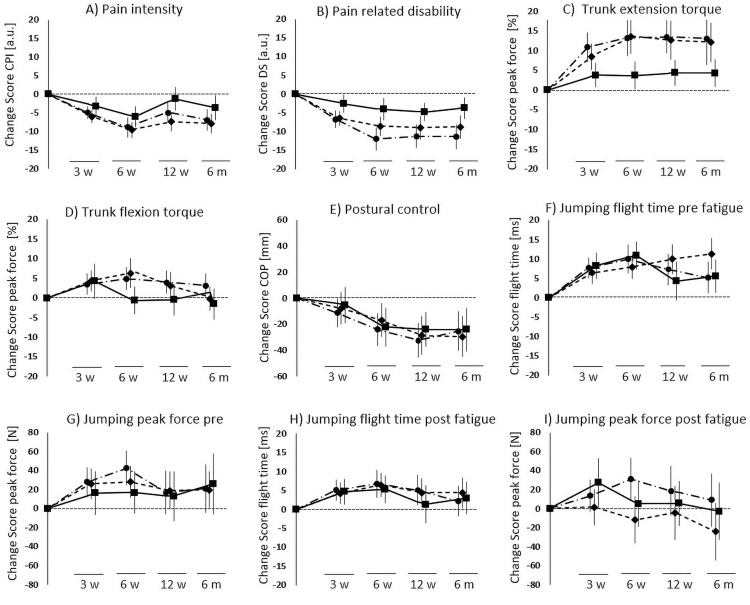
Gain scores of pain and functional outcomes overall assessment points. Displayed are the means ± 95% confidence interval, 12 w, after 12 Weeks; 3 w, after 3 weeks; 6 m, after 6 months; 6 w, after 6 weeks; CoP, center of pressure trace; CPI, characteristic pain intensity; dashed-dotted line, SMT + behavioral therapy; dotted line, SMT; DS, disability score; solid line, UCG.

The corresponding differences of functional outcomes revealed statistically significant differences for trunk torque (cumulated isometric and isokinetic testing, changes in percentage) during extension (ANOVA, F(2, 334)=4.247; *P*=.015) in both intervention groups compared to UCG (SMT, *P*=.045, d=0.38; SMT+BT, *P*=.019, d=0.44). No statistically significant difference between groups was found for trunk torque (cumulated isometric and isokinetic testing, changes in percentage) during flexion (ANOVA, F(2, 335)=1.582; *P*=.207), postural control (ANOVA, F(2, 301)=0.338; *P*=.714), CMJpre peak force (ANOVA, F(2, 275)=0.056; *P*=.946), CMJpre flight time (ANOVA, F(2, 341)=1.505; *P*=.223), CMJpost peak force (ANOVA, F(2, 258)=0.818; *P*=.442), and CMJpost flight time (ANOVA, F(2, 307)=0.799; *P*=.451) (fig 5). The gain scores of pain and functional outcomes for all assessment points normalized to baseline are presented descriptively in figure 6.

## Discussion

### Feasibility of the intervention

Considerably high adherence rates were found in both the sensorimotor exercise intervention and sensorimotor exercise combined with behavioral treatment intervention. In both groups, participants who maintained within the exercise program showed adherence rates of over 80%, which is commonly referred to as fully compliant.^14^^,^^18^ The dropout rates of both intervention groups during the center-based phase were also considerably low. However, the dropout rates of up to 50% at the end of the home-based intervention phase must be considered.

Adherence is generally often reduced in exercise treatments compared to cognitive behavioral interventions.^19^ However, in the current investigation, adherence rates did not differ between the 2 intervention groups because they both consisted of a major part of an exercise program. This may also be a reason for the considerably high dropout rates, especially in the self-administered home-based training phase in both intervention groups. Nondropout participants were training with a mean frequency of 2.5 sessions per week. Thus, the achieved training frequency falls slightly under a desirable amount of 3-5 sessions per week, which was previously found to be linked to the largest effects on pain and disability for sensorimotor/stabilization interventions.^13^

### Feasibility of the test setup

The conducted functional testing battery was identified as feasible, with only minor incidences where certain medical conditions did not allow the planned assessments. Despite being time-consuming, objective functional assessments are deemed to be important because neuromuscular alterations and impairments, interacting with psychological and social factors, are a contributor to both the onset and subsequent chronification of low back pain.^20^^,^^21^

### Effects of the interventions

Sensorimotor treatment and the combined sensorimotor and behavioral treatment were more effective than a usual care comparator group, based on the assessment from baseline to postintervention. Sensorimotor exercise alone showed positive effects for pain intensity and back extension torque, the combination with a behavioral treatment reduced pain-related disability and increased back extension torque. The positive effects of psychological interventions, such as (cognitive) behavioral therapy, delivered in combination with exercises have been proven before.^9^ However, the effect sizes of the current investigation were rather small, both in SMT and SMT+BT. It is further a matter of discussion, whether decisions on adopting psychosocial treatments should be undertaken on an individual basis, both to improve treatment effect and adherence.^22^ Such a stratified approach of individually prescribing the characteristics in combination with individualized prescription of psychological interventions (in particular behavioral-cognitive therapies) was found to be more effective than matched exercises alone.^22^^,^^23^ More detailed, the behavioral therapy components might be more helpful for those participants who display higher psychosocial risk factors (eg, yellow flags) despite nondifferent pain intensities.^24^

Apart from back extension torque, none of the other functional outcomes showed statistically significant intervention effects, in either group. In previous research, motor control deficits and functional alterations were found in low back pain, though findings were not always homogeneous among studies.^25^^,^^26^ Whether the absence of intervention effects of secondary functional outcomes in the present study is related to the absence of functional alterations in the investigated population (characterized by low to moderate pain), or whether the exercise program in general does not provoke changes in the assessed neuromuscular domains remains open.

### Clinical implications

To counteract dropouts and vice versa to improve adherence, careful coaching strategies are recommended.^27^ In accordance with the current findings, supervision and guidance, individualized interventions,^22^ self-management,^28^ and sophisticated information on approaches and potential effects^29^ may increase adherence rates.

Sensorimotor movement therapies, including often used specifications such as motor control,^5^ stabilization,^30^^,^^31^ functional restoration,^3^ and core-stability^32^ exercises, are likely to be the most effective active (exercise) treatments on low back pain^3^^,^^4^ and can, thus, be recommended. Derived from the current findings, sensorimotor exercises are effective in people with low back pain of low to moderate intensity. Scheduling such exercises 3 times a week with a duration of around 30 minutes leads to a mean training frequency of 2.5 times per week in this population. Additional psychological treatments are suggested to be relevant in those with a higher risk for chronification or worsening of the symptoms and those with high disability.^22^^,^^33^

### Study limitations

The results of the current study are representative for a population suffering from mild to moderate pain episodes. Although representing a vast majority of affected people, back pain patients with more severe clinical symptoms and functional limitations might yield different results. A short stretching session (of the extremities) was implemented as an activity to match the overall training session duration across intervention groups. We cannot rule out that this had no effect on the assessed outcome variables. Despite the randomized allocation, the between-group differences are present in the baseline values for age and pain. Conclusions drawn about the intervention effects are limited to the comparison from baseline to directly after the intervention (week 12). Descriptively provided information (fig 6) over all measurement time points (with follow-up) needs to be taken with caution because cumulating data loss over time did not allow for further statistical interpretation. Finally, the omission of dropouts from the analyses of intervention effects needs to be discussed as a limitation of this study.

## Conclusions

Sensorimotor exercise programs lead to high adherence rates but are prone to increased dropout rates during self-administered home-based training phases. Although sensorimotor training showed an increased effect on pain intensity, sensorimotor training in combination with behavioral intervention was more effective for disability. Both interventions led to increase in strength, indicative of a neuromuscular adaptation because of the sensorimotor exercise intervention. Effect sizes were rather small in the targeted sample of low to moderate levels of pain and disability.

Future studies are warranted, to derive individual target criteria to decide which patients may profit the most from SMT as single treatment or from SMT plus behavioral intervention. Therefore, motor control and psychosocial issues could be used as both stratification tool and intervention type setting.

## Suppliers

a. G*Power; Heinrich Heine University of Düsseldorf. b. Wii Balance Board; Nintendo. c. Humac Balance; CSMi Computer Sports Medicine. d. AMTI Force plate; AMTI Advanced Mechanical Technology. e. Kistler force plate; Kistler Instrumente. f. CON-TREX MJ/TP 1000; Physiomed. g. Isomed 2000; Ferstl. h. Tergumed Plus Flexion; Proxomed. i. Schnell m3 diagnos+; Schnell Trainingsgeräte. j. MATLAB-R2019; MathWorks. k. Excel 2016; Microsoft. l. SPSS, version 28.0; IBM.

## Disclosure

The investigators have no financial or nonfinancial disclosures to make in relation to this project.

## Ethical approval

Institutional ethics review board of the University of Potsdam (number 36/2011).

## Appendix Supplementary materials

Image, application 1
