# Supplementary material for: Sensorimotor Stabilization Exercises With and Without Behavioral Treatment in Low Back Pain: Feasibility and Effects of a Multicenter Randomized Controlled Trial
Source: Arch Rehabil Res Clin Transl. 2025 Jan 27;7(1):100430. doi: 10.1016/j.arrct.2025.100430 (PMC12128597; doi:10.1016/j.arrct.2025.100430)
Supplement: Supplementary file 1 [file mmc1.pdf]

PHOTO PUBLICATION RELEASE FORM

Publication title: Sensorimotor stabilization exercises with and without behavioural treatment in low back pain: Feasibility and effects of a multicentre randomized controlled trial

Researcher (First author): Tilman Engel

Contact Information: University of Potsdam, Am Neuen Palais 10, House 12, 14469 Potsdam, Germany

Consent of photo model:

I warrant that I am 18 years of age or older and hereby grant the researcher designated above from the University of Potsdam permission to use the pictures of myself (Training exercises (Figure_2)) as part of the above titled publication.

Printed Name: Josefine Stoll   Date: 22.11.2024

Signature: J. Stoll

Researcher:

Name: Tilman Engel   Date: 22.11.2024

Signature: Tilman Engel
